# Gender differences in disordered eating and weight dissatisfaction in Swiss adults: Which factors matter?

**DOI:** 10.1186/1471-2458-12-809

**Published:** 2012-09-20

**Authors:** Christine Forrester-Knauss, Elisabeth Zemp Stutz

**Affiliations:** 1 Swiss Tropical and Public Health Institute (Swiss TPH), Socinstrasse 57, Basel, Switzerland; 2 University of Basel, Petersplatz 1, Basel, Switzerland

## Abstract

**Background:**

Research results from large, national population-based studies investigating gender differences in weight dissatisfaction and disordered eating across the adult life span are still limited. Gender is a significant factor in relation to weight dissatisfaction and disordered eating. However, the reasons for gender differences in these conditions are still poorly understood. The aim of this study was to examine gender differences in weight dissatisfaction and disordered eating in the general Swiss adult population and to identify gender-specific risk factors.

**Methods:**

The study population consisted of 18156 Swiss adults who completed the population-based Swiss Health Survey 2007. Self-reported weight dissatisfaction, disordered eating and associated risk factors were assessed. In order to examine whether determinants of weight dissatisfaction and disordered eating (dieting to lose weight, binge eating, and irregular eating) differ in men and women, multivariate logistic regressions were applied separately for women and men.

**Results:**

Although more men than women were overweight, more women than men reported weight dissatisfaction. Weight category, smoking status, education, and physical activity were significantly associated with weight dissatisfaction in men and women. In women, nationality and age were also significant factors. Gender-specific risk factors such as physical activity or weight category were identified for specific disordered eating behaviours.

**Conclusions:**

The results suggest that gender specific associations between predictors and disordered eating behaviour should be considered in the development of effective prevention programs against disordered eating.

## Background

Disordered eating and the increasing prevalence of obesity in Western societies are major public health concerns [1,2]. These conditions are strongly related to gender [3-5], but the reasons underlying the observed gender differences are poorly understood. Disordered eating, defined as harmful and often ineffective eating behaviours used with the aim of losing weight or changing one’s appearance [6], was reported by nearly a third of the Swiss population [7], and has been shown to predict weight gain and to be a risk factor for the onset of obesity [8] and eating disorders [9]. Dissatisfaction with one’s own body weight is an attitudinal component of body image [10], which predicts dieting behaviour [11], stress, depression, bulimic symptoms and low self-esteem [12]. Weight dissatisfaction, with prevalence rates ranging from a third to more than one half of the population [7,13] was associated with disordered eating in cross-sectional studies [14] and identified as a risk factor for the development of disordered eating in longitudinal studies [15,16].

Gender is known to play an important role in relation to weight dissatisfaction and disordered eating behaviours [3,17]. Although the prevalence of obesity in adulthood was higher in men, women showed greater dissatisfaction with their weight than men [7,13,18], dieted more frequently, and demonstrated more disordered eating than men, particularly in younger adulthood [18,19]. This has also been found in the Swiss Health Survey 2007 [7]. Research about these problems has often focused on young women [4,20,21]. Weight dissatisfaction seems to be prevalent across the entire life span in women. It was even found to be one of the most important body image concerns in women aged 61–92 years [22]. Body dissatisfaction has been shown to be stable in adult women [21,23]. In contrast, in men the desire to lose weight has been found to increase as they get older [4].

Previous studies have reported inconsistent results relating to gender differences associated with the relationship between BMI and weight dissatisfaction: Tiggemann [5] has indicated a weaker association between BMI and body dissatisfaction in women compared to men, while Millstein et al. [13] suggested similar associations for both genders. It has been found that in both genders a higher BMI was associated with the engagement in dieting behaviours [24]. In boys, weight perception and dieting have been significantly associated with BMI [20] and a higher BMI was associated with greater body dissatisfaction and a greater likelihood of changing eating habits [3,25]. Women often wish to lose weight, whereas men want a body that is either larger and more muscular, or thinner [4,25,26]. However, the extent to which BMI is related to weight dissatisfaction in adult men has not been fully investigated [4,20] and the gender difference in this association is not fully clear.

The prevalence of dieting is higher in women than in men [19,27,28]. Dieting was even found to be prevalent in older age groups [5]. In a population-based Swiss study 42 % of the 30–74 year-old women had dieted in the last 5 years [29]. Of those, 67 % had a normal weight. Of the women older than 67 years of age, 31 % had dieted, although 62 % of them had a normal weight. In a study by Tiggemann and Lynch [23] disordered eating in women was found to decline with increasing age.

Other eating behaviours, such as binge eating, appear to have similar prevalence rates in men and women [30,31]. In the study by Lavender et al. [30] the results of different studies were compared and showed that 25 % of the men reported binge episodes, 24 % dietary restraint and 3.2 % self-induced vomiting [30]. Women had comparable prevalence rates [30,32]. Other studies have found higher binge eating rates in women than in men [28,33].

It has been suggested that physical activity might be a protective factor in the development of disordered eating behaviours, particularly in girls [27,34]. Results of a meta-analysis showed that the risk of disordered eating was reduced in non-elite athletes compared to controls [34]. However, under certain circumstances, depending on the type of exercise and the reason for exercise, sports participation can also be a risk factor for disordered eating [34-36]. In a representative sample of Canadian men, daily physical activity was a risk factor for eating disorders [37].

A relationship between gender, weight dissatisfaction and disordered eating has been indicated [3,38]. However, results from population-based studies investigating gender differences in specific disordered eating behaviours and gender specific risk factors are still needed [17]. Furthermore, the understanding of weight dissatisfaction and disordered eating across the adult life span in men and women is still limited [21].

The aim of the current study was to examine the prevalence rates of both, weight dissatisfaction and disordered eating (such as dieting, binge eating and irregular eating) in a large representative sample of Swiss adults. Furthermore, the association of weight dissatisfaction and disordered eating with sociodemographic factors (education, age, nationality), and with weight category, smoking and physical activity in men and women was examined.

## Methods

Data presented in this paper were taken from the Swiss Health Survey (SHS) 2007, a nationwide, population-based Swiss study conducted every five years since 1992 by the Swiss Federal Office of Statistics. The SHS collects detailed information on health status, health-related behaviours, socioeconomic factors, and health care utilization. A random sample is drawn from individuals living in Switzerland aged 15 years or older. It is based on two national laws of statistical assessments 1) from 30th June 1993 (SR 431.012.1), and 2) the law of the national census of the population from the 19th December 2008 (SR 431.112.1). Participants had to be able to speak at least one of three interview languages (German, French or Italian). Subjects were asked to participate in a telephone interview and then to complete a written questionnaire. The response rate for the telephone interview was 66 % (8,424 men and 10,336 women). Of these, 77 % returned the written questionnaire (6,308 men and 8,085 women). Data was weighted by age, sex and nationality to assure representativeness of the Swiss population. Some questions relating to weight dissatisfaction and disordered eating were only asked to participants younger than 50 years of age because this age group was considered to be of particular interest in relation to weight dissatisfaction and disordered eating. The items on weight dissatisfaction had been used in a previous national sample [39]. There was no ethics approval necessary in Switzerland for studies by the Swiss Federal Office of Statistics.

### Participants

This study only included data from Swiss adults (those aged over 18: N = 18,156). Mean age was 50.7 years (men: x = 49.60, SD = 17.28; women: x = 51.56, SD = 18.02). The sample consisted of 55.4 % women, 87.0 % of the sample were of Swiss nationality.

### Measures

#### Weight dissatisfaction

For the assessment of weight dissatisfaction participants were asked “Are you satisfied with your body weight at the moment?” with the response options “totally satisfied”, “quite satisfied”, “not quite satisfied”, and “not satisfied at all”. Only participants younger than 50 years of age were additionally asked if they would like to change their weight, with the response options yes/no, and, in the case of a positive answer, which of the following statements were true for them: “I would like to lose weight, but that is not my biggest worry”, “I would like to lose weight and I think about it all the time”, “I would like to gain weight, but this is not my biggest worry”, and “I would like to gain weight and I think about it all the time”.

### Disordered eating

In the Swiss Health Survey 2007, only participants younger than 50 years of age were asked questions about their disordered eating behaviour because this age group was of particular interest in relation to disordered eating. Dieting was assessed with two items: Participants were first asked if they had gone on a diet in the preceding 12 months. If they answered with “yes” they were asked whether that was “to lose weight, without any medical reasons”, “for medical reasons”, or “for other reasons (neither medical, nor to lose weight)”. Binge eating and self-induced vomiting were assessed by asking participants if within the last few months they had eaten a large amount of food and could not stop eating, or if they had made themselves vomit. Irregular eating was assessed by asking participants if they had eaten irregularly within the last 12 months (not had had fixed meal times).

### Body mass index

Body mass index (BMI) was calculated from self-reported weight and height (kg/m^2^). Underweight was defined as a BMI below 18.5, normal weight as a BMI between 18.5 and below 25, overweight as a BMI between 25 and below 30, and obese as a BMI of 30 or above.

### Smoking status and physical activity

The study assessed smoking, former smoking or non-smoking with the questions, “Do you smoke, even if only occasionally?” with ‘yes’ and ‘no’ response options, and “Have you smoked regularly for more than 6 months?” (yes/no).

Physical activity was assessed by asking participants, “In your free time, do you exercise until you sweat, at least once per week? For example by walking fast, running or bike riding?”, and “On how many days per week on average?” Participants were also asked on how many days per week they were physically active to the extent that they got out of breath, for example through fast walking, hiking, dancing, working in the garden or many types of sports, and for how many hours and minutes they were on average active in this way. Participants were classified as inactive, partly active, and active depending on the number of times they had sweated and the length of time or number of times they had been out of breath due to physical activity per week based on the classification used in previous Swiss Health Survey analysis [40].

### Statistical analysis

Analyses were conducted using STATA 10. Weighted prevalence rates of weight category, weight dissatisfaction, and disordered eating were calculated for men and women separately. Due to the oversampling of some population segments (e.g. Italian language region), prevalence rates were weighted for the Swiss population, taking into account the particularities of the sampling strategy with regards to age, sex, nationality and language region [41]. In order to assess whether determinants of weight dissatisfaction and disordered eating (dieting to lose weight, binge eating, and irregular eating) differ between men and women, multivariate logistic regressions were conducted separately for women and men. This analytic approach has been proposed in order to identify whether different factors matter in men and women, or whether associations differ in direction and strength [42,43]. The statistical analyses were considered to be significant with p < .05.

## Results

### Prevalence of weight category, weight dissatisfaction, and disordered eating

Mean body mass index was 25.4 (SD = 3.63) for men and 23.7 (SD = 4.29) for women. Men had a significantly higher body mass index than women (t (10365) = 19.56, p < .001). The percentage of overweight men was nearly twice as high as that for women (see Table 1). More women (5.6 %) than men (0.8 %) were underweight. Weight dissatisfaction was reported by 36.7 % of the women, and 29.5 % of the men. Over half of the women (55.7 %) and half of the men (50.1 %) wanted to change their weight; mostly wanting to lose weight. However, 17.7 % of the men wished to gain weight, compared to 6.9 % of the women. Of the women, 11.2 % and of the men 6.9 % reported that they had dieted in the last 12 months. Losing weight was reported as the reason for dieting in over half of the women who reported dieting, and in 40.6 % of men. Irregular eating was the most prevalent disordered eating behaviour; followed by dieting, binge eating and self-induced vomiting. Irregular eating and binge eating were equally prevalent in men and women. Self-induced vomiting was a rarely reported disordered eating behaviour.

**Table 1:** Weighted prevalence of weight category, weight dissatisfaction, and disordered eating in Swiss women and men

	**Women %**	**Men %**
*Weight category*		
Underweight (BMI < 18.5)	5.6	0.8
Normal weight (BMI 18.5 - <25)	64.6	50.7
Overweight (BMI 25 - <30)	21.7	39.5
Obese (BMI > =30)	8.1	9.0
Total	100.00	100.00
*Weight dissatisfaction*		
Very satisfied	30.8	38.3
Quite satisfied	31.9	32.2
Not quite satisfied	27.7	24.9
Not satisfied at all	9.7	4.6
Total	100.00	100.00
Wish to change weight*		
Want to change weight	55.7	50.1
Do not want to change weight	44.3	49.7
Total	100.00	100.00
Want to gain weight	6.9	17.7
Want to lose weight	93.1	82.3
Total	100	100
*Disordered eating*		
Dieting in the last 12 months	11.2	6.9
Dieting to lose weight**	54.26	40.59
Irregular eating*	19.2	20.0
Binge eating (every day – 1 to several times per week)*	5.3	5.8
Self-induced vomiting (every day – 1 to several times per week)*	0.3	0.2

*These questions were only asked of participants under 50 years of age; ** of those that reported dieting.

### Predictors of weight dissatisfaction

In men, weight dissatisfaction was associated with weight category, smoking status, education and physical activity (see Table 2). Underweight (OR = 5.86, p < .001), overweight (OR = 6.44, p < .001) and obese (OR = 19.05, p < .001) men showed significantly higher odds of being dissatisfied with their weight than normal weight men. Men who used to smoke were significantly more likely to report weight dissatisfaction than non-smokers. Men with higher education had a significantly higher likelihood of reporting weight dissatisfaction compared to men with only compulsory school education (secondary school: OR = 1.36, p < .01; college/university: OR = 1.99, p < .001). Men who reported being partly physically active showed a significantly higher likelihood of being dissatisfied with their weight than men who were physically inactive (OR = 1.20, p < .05).

**Table 2:** Predictors of weight dissatisfaction in women and men

	**Weight dissatisfaction**			
	**Men (n = 7570)**		**Women (n = 9516)**	
	**OR**	**(95%CI)**	**OR**	**(95%CI)**
**Weight category**^**Δ**^				
Normal weight	1		1	
Underweight	5.86***	(3.19-10.76)	0.71**	(0.56-0.90)
Overweight	6.44***	(5.66-7.33)	6.91***	(6.17-7.74)
Obese	19.05***	(15.64-23.21)	15.97***	(13.21-19.29)
**Age**				
18-24	1		1	
25-34	1.13	(0.84-1.53)	1.02	(0.82-1.28)
35-44	1.19	(0.90-1.58)	0.87	(0.70-1.07)
45+	0.93	(0.71-1.22)	0.71**	(0.58-0.87)
**Nationality**				
Swiss	1		1	
Other nationalities	1.12	(0.95-1.31)	1.34***	(1.15-1.56)
**Smoking**				
Non-smoker	1		1	
Former smoker	1.24**	(1.08-1.43)	1.57***	(1.39-1.77)
Smoker	1.01	(0.89-1.16)	1.28***	(1.34-1.44)
**Education**				
Compulsory schooling	1		1	
Secondary school	1.36**	(1.09-1.68)	1.51***	(1.32-1.75)
College/university	1.99***	(1.59-2.49)	1.50***	(1.27-1.78)
**Physical activity**				
Inactive	1		1	
Partly active	1.20*	(1.02-1.42)	1.32***	(1.16-1.51)
Active	0.90	(0.76-1.07)	1.16*	(1.01-1.33)

* = p < .05; ** = p < .01, *** = p < .001; Δ Normal weight = BMI 18.5 - <25, Underweight = BMI < 18.5, Overweight = BMI =25 - <30, Obese = BMI > =30.

In women, weight dissatisfaction was associated with weight category, age, nationality, smoking status, education and physical activity (see Table 2). While underweight women were less likely to report weight dissatisfaction (OR = 0.71, p < .01), overweight (OR = 6.91, p < .001) and obese (OR = 15.97, p < .001) women had significantly higher odds of being dissatisfied with their weight than normal weight women. Women over the age of 45 were less likely to report weight dissatisfaction than women between 18 and 24 years of age (OR = 0.17, p < .01). Non-Swiss women were more likely to report weight dissatisfaction than Swiss women (OR = 1.33, p < .001). Smokers and former smokers, as well as women with a higher education, were also more likely to report weight dissatisfaction. Partly physically active (OR = 1.32, p < .001) and physically active (OR = 1.17, p < .05) women had higher odds of being dissatisfied with their weight.

### Predictors of dieting, binge eating, and irregular eating

Reporting disordered eating behaviour (dieting, binge eating and irregular eating) was significantly more likely in women and men with higher weight dissatisfaction (see Table 3). This was the strongest association compared to the other observed associations. The likelihood of dieting was significantly higher in overweight (OR = 2.15, p < .01) and obese (OR = 3.00, p < .01) men than in normal weight men and in partly physically active (OR = 2.00, p < .05) or physically active (OR = 2.62, p < .01) men than in physically inactive men. The odds for binge eating were lower for men over the age of 35 than for younger men and for men with higher education. The likelihood of irregular eating was higher in men with a non-Swiss nationality, in smokers than in non-smokers, and lower in men older than 18–24 years.

**Table 3:** Predictors of dieting, binge eating, and irregular eating in women and men

	**Dieting to lose weight**				**Binge eating**				**Irregular eating**			
	**Men (n = 515)**		**Women (n = 1068)**		**Men (n = 4016)**		**Women (n = 4603)**		**Men (n = 4017)**		**Women (n = 4602)**	
	**OR (95%CI)**		**OR (95%CI)**		**OR (95%CI)**		**OR (95%CI)**		**OR (95%CI)**		**OR (95%CI)**	
**Weight dissatisfaction**	2.35***	(1.53-3.60)	3.30***	(2.43-4.48)	2.18***	(1.59-2.98)	2.68***	(2.01-3.57)	1.47***	(1.21-1.78)	1.45***	(1.22-1.72)
**Weight category**^**Δ**^												
Normal weight	1		1		1		1		1			
Underweight	+	+	0.05***	(0.01-0.23)	2.14	(0.72-6.39)	0.90	(0.50-1.61)	1.86	(0.88-3.96)	1.04	(0.78-1.39)
Overweight	2.15**	(1.35-3.42)	1.21	(0.86-1.70)	1.25	(0.90-1.73)	1.00	(0.70-1.43)	1.07	(0.88-1.29)	1.06	(0.85-1.34)
Obese	3.00**	(1.54-5.85)	0.73	(0.48-1.10)	1.59	(0.96-2.62)	1.80**	(1.18-2.74)	1.29	(0.93-1.80)	1.23	(0.89-1.70)
**Nationality**												
Swiss	1		1		1		1		1			
Other nationality	0.65	(0.37-1.12)	1.66*	(1.09-2.52)	0.88	(0.61-1.28)	1.26	(0.91-1.75)	1.37**	(1.12-1.70)	1.51***	(1.24-1.85)
**Smoking**												
Non-smoker	1		1		1		1		1		1	
Former smoker	0.96	(0.61-1.52)	1.34	(0.97-1.87)	0.96	(0.64-1.43)	1.07	(0.76-1.51)	1.14	(0.89-1.46)	0.94	(0.74-1.19)
Smoker	1.57	(0.96-2.57)	0.92	(0.66-1.29)	0.88	(0.65-1.20)	0.74	(0.55-1.00)	1.91***	(1.60-2.28)	2.11***	(1.79-2.49)
**Age**												
18-24	1		1		1		1		1			
25-34	0.60	(0.21-1.71)	1.01	(0.51-2.00)	0.94	(0.61-1.44)	0.93	(0.62-1.40)	0.71*	(0.55-0.92)	0.72**	(0.57-0.92)
35-44	1.24	(0.47-3.30)	0.78	(0.41-1.49)	0.55**	(0.36-0.85)	0.62*	(0.42-0.93)	0.63***	(0.49-0.80)	0.47***	(0.37-0.59)
45+	0.57	(0.22-1.45)	0.45*	(0.24-0.84)	0.47**	(0.28-0.79)	0.50**	(0.30-0.82)	0.50***	(0.37-0.67)	0.56***	(0.42-0.73)
**Education**												
Compulsory schooling	1		1		1		1		1		1	
Secondary school	1.03	(0.45-2.38)	1.53*	(1.02-2.30)	0.45**	(0.27-0.73)	0.56**	(0.37-0.85)	1.03	(0.72-1.47)	1.03	(0.77-1.39)
College/university	1.53	(0.65-3.57)	1.00	(0.64-1.58)	0.32***	(0.19-0.55)	0.45**	(0.28-0.72)	0.71	(0.49-1.03)	1.00	(0.72-1.37)
**Physical activity**												
Inactive	1		1		1		1		1		1	
Partly active	2.00*	(1.04-3.85)	1.82**	(1.23-2.68)	0.81	(0.51-1.26)	0.91	(0.62-1.33)	1.10	(0.84-1.44)	0.63***	(0.51-0.78)
Active	2.62**	(1.37-5.02)	1.49*	(1.02-2.19)	1.05	(0.67-1.64)	1.01	(0.69-1.49)	1.26	(0.96-1.66)	0.73**	(0.58-0.91)

* = p < .05; ** = p < .01, *** = p < .001; + = not enough cases to perform the calculation; OR = Odds ratios, CI = Confidence interval; **Δ** Normal weight = BMI 18.5 - <25, Underweight = BMI < 18.5, Overweight = BMI =25 - <30, Obese = BMI > =30.

Underweight women were significantly less likely to report dieting behaviour (OR = 0.05, p < .001) than normal weight women, women with non-Swiss nationality more likely to diet than Swiss women (OR = 1.66, p < .05), and former smokers more likely to report dieting than non-smokers. Women over the age of 45 were less likely to report dieting than women aged between 18 and 24 years (OR = 0.45, p < .05). Women with secondary education compared to only compulsory education, and partly physically active (OR = 1.82, p < .01) and physically active women (OR = 1.49, p < .05) compared to inactive women were more likely to diet. The odds for binge eating were higher in obese women than in normal weight women and lower in women older than 35 years of age and with an educational level above compulsory education. The likelihood of irregular eating was higher in women with non-Swiss nationality and smokers, and lower in women over the age of 25 and in physically active women.

## Discussion

This Swiss population-based study showed that although more men than women were overweight, more women than men reported being dissatisfied with their weight, wanting to change their weight and having dieted within the last 12 months. No gender difference was found for the prevalence of irregular eating and binge eating. While some predictors of weight dissatisfaction and disordered eating were similar for men and women, others were significant only for one gender, and for a few associations the direction was discrepant.

### The prevalence of weight dissatisfaction and disordered eating

The prevalence of weight dissatisfaction in this population-based study of Swiss adults was higher than the prevalence found by Millstein et al. [13] in their population-based study from the US, but lower than in an earlier Swiss study with middle-aged and older women [29], and slightly lower than found in an Icelandic population-based study [38]. It remains unclear whether these differences are due to differences in the study population, the use of different measures, or whether weight dissatisfaction has decreased in Switzerland since the 1990s, which is a period when overweight has increasingly been addressed as a public health topic. It has been shown that prevalence of overweight individuals did not significantly increase between 2002 and 2007 in Switzerland [7], which might have had an effect on weight dissatisfaction in the Swiss population. Longitudinal studies investigating changes in BMI and weight dissatisfaction are needed in order to understand the effects of BMI on weight dissatisfaction over time. Higher prevalence rates of disordered eating behaviours in women than in men were found in earlier studies [19,27,28,33]. This was observed in the present study only for dieting, whereas irregular eating and binge eating were no more prevalent in women than in men, a finding also recently reported by Lavender in undergraduates [30].

### Predictors of weight dissatisfaction

Being overweight and being obese were the strongest predictors of weight dissatisfaction in both men and women, whereas discrepant directions of associations were found for being underweight (a risk factor in men but a protective factor in women). In men, weight dissatisfaction was more likely in underweight as well as in overweight or obese men, which was consistent with the results of other studies [4,25,26,44], but contrasts with results from Tiggemann [5], who suggested BMI is a similarly important factor in weight dissatisfaction for both women and men. These results are consistent with the hypothesis that weight dissatisfaction in men and women are related to the thin body ideal for women and the muscular body ideal for men as portrayed by media. Being of non-Swiss nationality, aged above 45 years, current smoking and the highest level of physical activity were associated with weight dissatisfaction in women only. Age was not a significant predictor of weight dissatisfaction in men. Women over the age of 45 were less likely to report weight dissatisfaction than younger women. Contradicting results were found in a previous study with weight dissatisfaction being not only reported by younger but also by older women [22]. The higher likelihood of being satisfied with one’s weight at an older age in this study might be due either to generational effects or to a higher acceptance of one’s own body. Generational effects might be due to the confrontation to different body ideals portrayed by media in earlier times. Weight and the comparison to mostly young body ideals as portrayed in media might be less important to older women.

As found in earlier studies [13,29,38], weight dissatisfaction was significantly associated with education. Individuals with higher levels of education reported more weight dissatisfaction than individuals with lower education. This was found for both genders*.* It might be that the current thin body ideal for women and muscular body ideal for men is more internalised by individuals with a higher education.

Former smokers were significantly more likely to be dissatisfied with their weight than non-smokers. This could be related to weight gain after smoking cessation. Interestingly, partly active individuals were more likely to report weight dissatisfaction than inactive individuals. In women, active individuals were also more likely to report weight dissatisfaction than inactive individuals. In this study the reasons for exercising such as for health, fitness, enjoyment or weight control were not assessed. These reasons for exercising might play a role in relation to weight dissatisfaction. Tiggemann and Williamson [45] have shown that the desire to change weight and tone one’s body was more often a reason for exercising in women than in men. Furthermore, exercising to change one’s weight was significantly related to body dissatisfaction. Thus, the reasons for exercising might mediate the relationship between physical activity and weight dissatisfaction. Exercise type, which has not been assessed in this study, has also shown to play a role in relation to body dissatisfaction [35] and might be a further relevant explaining factor.

### Predictors of disordered eating

The pattern of predictors of disordered eating behaviours was more heterogeneous, particularly for dieting. Weight dissatisfaction consistently predicted all three forms of disordered eating in both men and women and this was particularly pronounced for dieting and binge eating in women. This is consistent with earlier results [14]. Interestingly, overweight or obese women were not significantly more likely to report dieting or irregular eating, and being underweight was only in women negatively associated with dieting. However, overweight or obese men were significantly more likely to report dieting than normal weight men. Therefore, disordered eating in women seems to be related to a perceived and not an actual overweight, while dieting in men seems to be related to actual weight. It has been shown earlier, that women overestimated their weight, even if they had a low BMI. Men underestimated their weight and therefore had a lower likelihood to try to lose weight [46]. Binge eating was less likely in older individuals and in individuals with a higher education in both men and women. Predictors were also quite similar for irregular eating in men and women. Smoking might be an indicator for higher health risk behaviours in other areas such as disordered eating. Older age was a protective factor against binge eating and irregular eating in men and women. It may be that the older generation is more used to having regular mealtimes. This is consistent with the results of Tiggemann and Lynch [23], who showed a decline in disordered eating for women of an older age, and with results of a population-based sample which showed that binge eating was negatively associated with age [47]. Furthermore, being of non-Swiss nationality, aged above 45 years, and having a secondary education was associated with dieting to lose weight only in women. Being partly physically active or active was protective against irregular eating in women, no association was found in men, and it was a risk factor for dieting in both men and women. It was not significantly associated with binge eating behaviour. Therefore, physical activity cannot be seen as a general protective factor for eating problems, as suggested by Rosendahl et al. [27], but might play a specific role in relation to dieting behaviour. Dieting behaviour in individuals who are physically active might be used to enhance athletic performance, or might be used to try to change one’s body size and shape.

### Limitations of the study

The limitations of this study include the cross-sectional design, which does not allow conclusions about causality. Some questions were only asked of participants under 50 years of age and no data exists of individuals older than 50. Therefore, in all tables the sample sizes have been reported. As the aim of the study was to assess health and health behaviour in a representative sample of Swiss adults, brief self-report measures were used to assess weight dissatisfaction and disordered eating. These measures are more likely to lead to misclassification than more elaborate indices, and they do not have the same explanatory power. However, the strength of this study is that it uses a large, population-based, representative sample. Data was assessed in 2007 and therefore has to be interpreted as valid for this time period.

## Conclusions

Given the different pattern of prevalence and predictors of weight dissatisfaction and disordered eating behaviours in adult men and women, an understanding of these related factors is important for the development of gender-appropriate prevention or intervention programs to reduce disordered eating and to prevent weight gain.

The results of the current study showed that weight dissatisfaction and dieting to lose weight were more common in women, whereas binge eating and irregular eating were similarly common among both genders. Similar and different predictors of weight dissatisfaction and of disordered eating behaviours were found for both genders. For the prevention of weight dissatisfaction, attention should be paid to overweight and obesity in men and women. In women, underweight, smoking, ethnicity and physical activity are also predictors that should be taken into consideration in the prevention of weight dissatisfaction. For the prevention of disordered eating behaviour, weight dissatisfaction seems to be a significant predictor in both genders. However, dieting will call for gender-specific prevention.

## Competing interests

No competing interests exist.

## Authors’ contributions

First author CFK performed the statistical analysis, did the literature searches, and wrote the manuscript. Author EZS contributed to the interpretation of the results, and the writing.

## Pre-publication history

The pre-publication history for this paper can be accessed here:

http://www.biomedcentral.com/1471-2458/12/809/prepub
